# Cardiovascular Disease Risk Assessment: Triglyceride/High-Density Lipoprotein versus Metabolic Syndrome Criteria

**Published:** 2019-05-13

**Authors:** Mojgan Gharipour, Masoumeh Sadeghi, Pouya Nezafati, Minoo Dianatkhah, Nizal Sarrafzadegan

**Affiliations:** ^1^Isfahan Cardiovascular Research Center, Cardiovascular Research Institute, Isfahan University of Medical Sciences, Isfahan, Iran; ^2^Cardiac Rehabilitation Research Center, Cardiovascular Research Institute, Isfahan University of Medical Sciences, Isfahan, Iran; ^3^Hypertension Research Center, Cardiovascular Research Institute, Isfahan University of Medical Sciences, Isfahan, Iran; ^4^Heart Failure Research Center, Cardiovascular Research Institute, Isfahan University of Medical Sciences, Isfahan, Iran

**Keywords:** Cardiovascular disease, Risk assessment, Triglyceride, High-density lipoprotein, Metabolic syndrome

## Abstract

**Background:** As finding subjects at risk of cardiovascular diseases based on the presence of metabolic syndrome (MetS) is time-consuming for physicians, we aimed to compare the effectiveness of triglyceride (TG)/high-density lipoprotein cholesterol (HDL-C) compared to MetS criteria in identifying high-risk individuals.

**Study design:** A prospective cohort study.

**Methods:** Isfahan cohort study was a longitudinal population-based study conducted on adults aged 35 yr or older, living in three districts in central part of Iran from Jan 2, 2001 to Sep 28, 2001. After 10 years of follow-up, participants were re-evaluated. The hazard ratio (HR) for cardiovascular disease events based on TG/HDL-C, sex-specific cut-off points, and MetS were also estimated. Akaike’s information criteria (AIC) were used as indicators of the goodness of fit of the model and prediction error.

**Results:** TG/HDL-C alternate cut-off points of 3.76 and 4.42 had a strong predictive value for CVD events but did not perform as well as MetS criteria. The unadjusted HR was greatest in the high-risk individuals by the MetS criteria (HR=2.08, 95% CI: 1.80, 2.41) compared to those identified as high-risks by the TG/HDL cut-off points and continued to be greatest after adjustments in different models. Based on the AIC, the best model is adjusted for sex, age, diabetes, total cholesterol levels, current smoker, diet, physical activity, and BMI.

**Conclusion:** MetS criteria appears to be a superior marker compared to TC/HDL-C to identify patients at cardiovascular risk, though lipid ratio also shows a remarkable predictive value and could be considered to achieve this goal when appropriate.

## Introduction

Metabolic syndrome (MetS) is considered a major health problem that directly increases the risk of developing atherosclerotic coronary heart disease^1^. Patients with MetS are also at increased risk of developing type 2 diabetes mellitus^2^. MetS can be used to predict if subjects are at risk of severe cardiovascular endpoint^3^. It increases the risk of cardiovascular disease (CVD) by approximately 2.33 folds among the Iranian population^4^.

According to the National Cholesterol Education Program (NCEP) Adult Treatment Panel (ATP) III^5^, patients with MetS criteria are defined as if he/she has three or more of the following characteristics: (hypertension, hypertriglyceridemia, lowered HDL-C, hyperglycemia, and central obesity). As finding at-risk subjects based on the presence of MetS is time-consuming for physicians, several expert groups have attempted to establish simple diagnostic criteria to use in clinical practice to identify patients who manifest the multiple components of MetS. The ability to identify these high-risk individuals before CVD manifests would be a significant clinical advantage, and the possibility that the plasma concentration ratio of triglyceride (TG)/high-density lipoprotein cholesterol (HDL-C) might fulfill this function has been raised^6,7^.

Using the plasma triglyceride/high-density lipoprotein cholesterol (TG/HDL-C) concentration ratio to predict cardiovascular events is as effective as using MetS, but the cut-off of the TG/HDL-C ratios differs among various populations because genetic and lifestyle factors have a great impact^8^.

Recently, we determined a specific TG/HDL-C cut-off point for the Iranian population with the following values: women >3.76 and men >4.42^9^. Those values are higher than the previous TG/HDL-C cut-off points that were observed in other populations [women: ≥2.5 and men ≥3.5], and they provide useful cut-off points for identifying individuals who meet the MetS criteria and who are at increased cardiometabolic risk^10^.

Therefore, we aimed to compare the Iranian TG/HDL-C ratio cut-off point with the MetS criteria to determine the effectiveness of each in predicting CVD events among an Iranian population.

## Methods

### Study population

The Isfahan Cohort Study (ICS) is a population-based, ongoing longitudinal study of adults aged 35 yr or more, living in urban and rural areas in three counties in central Iran: Isfahan, Najafabad and Arak^11^. Participants were recruited from Jan 2, 2001 to Sep 28, 2001. The participants were selected by multistage random sampling and they were recruited to reflect the age, sex, and urban/rural distribution of the community^11^.

The Ethics Committee of the Isfahan Cardiovascular Research Institute approved the study.

### Assessments

After obtaining informed written consent from each participant, a medical interview and physical examination were conducted. Blood pressure and anthropometric parameters were measured and fasting blood tests were carried out following standard protocols and using calibrated instruments ^12,13^. Subjects who smoked daily were considered to be “current smokers”. Waist circumference (WC) was taken as the smallest circumference at or below the costal margin. Hypertension was defined as systolic blood pressure (SBP) ≥140 mm Hg or diastolic blood pressure (DBP) ≥90 mm Hg in men and women, or as treatment of previously diagnosed hypertension^14^. Diabetes mellitus was defined as hyperglycaemia at more than 126 mg/dl fasting blood sugar (or use of diabetes medications). Physical activity was expressed as metabolic equivalent task in minutes per week and was obtained through an oral questionnaire. For calculating the dietary diversity score (DDS), the sum of the diversity scores of the six food groups (grain diversity score, dairy diversity score, fruit diversity score, vegetable diversity score, meat diversity score and oil diversity score) was considered^15^.

All determinations of lipids and lipoprotein cholesterol concentrations were performed in the Isfahan Cardiovascular Research Center Laboratory. Plasma total cholesterol (TC) and triglyceride (TG) levels were analyzed with a Hitachi analyzer (Japan) using enzymatic reagents (Pars Azmon, Tehran, Iran). High-density lipoprotein cholesterol (HDL-C) was measured in the supernatant fraction. Low-density lipoprotein cholesterol (LDL-C) was calculated using the Fried Ewald Formula^16^ If the TG levels were greater than 400 mg/dl LDL, cholesterol was measured directly.

Participants were evaluated according to the Mets criteria of the National Cholesterol Education Program (NCEP) Adult Treatment Panel (ATP) III^17^. To be classified as having MetS, participants must have exhibited three or more of the following characteristics: abdominal obesity, measured by WC (WC ≥102 cm in men and ≥ 88 cm in women); high blood pressure (systolic BP ≥130 mmHg or diastolic BP ≥85 mmHg); triglycerides (TG) ≥150 mg/dL; high-density lipoprotein-cholesterol (HDL-C) <40 mg/dL in men and <50 mg/dL in women; and fasting blood sugar (FBS) ≥110 mg/dL^5^.

### Follow-up surveys

After the baseline survey in 2001, follow-up of the participants was conducted every two years. Telephone interviews were conducted in 2003 and again in 2005–2006. A third telephone interview follow-up was completed in 2011. The participants, or their close family members, were asked about the participant’s health status via a questionnaire with a specific focus on cardiovascular and cerebrovascular events. The participants were asked if they had experienced any of the following neurological symptoms within the previous two years: hemiparesis, dysarthria, facial asymmetry, imbalance, and transient monocular blindness; and the following nine cardiovascular symptoms: central chest pain, chest discomfort, radiating pain or numbness (arm, shoulder, neck, or jaw), feeling dizzy or light-headed, loss of consciousness, nausea or vomiting, fever or sweating, shortness of breath, and any other symptom or pain. They were questioned more in detail if they had experienced any of those conditions. Second and third phone call with a more experienced nurse and physician, respectively, were made if neurological and cardiovascular panels found it necessary. In the last two phone calls, the subjects were asked open-ended questions to obtain more detailed descriptions about the event, such as the time of its occurrence and duration, additional symptoms, and changes in the activities of daily livings due to the event. Moreover, if any possible hospitalization had occurred, hospital records were found and summarized by experienced personnel and were reviewed by a cardiac panel and a neurological panel.

If a participant died, a death scenario was requested, and the patient was assumed to have died because of CVD only if other etiologies, such as a motor vehicle accident, could be ruled out. The verbal autopsy used a pre-defined questionnaire including questions about the participant’s medical history and the signs and symptoms before death.

Overall, 85.9% (5430 subjects) of the initial 6323 participants could be surveyed again and entered our study to obtain information concerning incidences of CVD events. Among the 5430 subjects enrolled in our study, both fatal and non-fatal CVDs were considered as CVD events.

### Statistical analysis

Data entry was carried out using EPI Info^TM^. The data were analyzed using STATA software (Stata/IC 11.0, StataCorp LP, College Station, TX, USA). For all analyses, statistical significance was assessed at the level of 0.05 (two-tailed). No variable had more than 3% missing values. Stochastic regression was used to impute the missing values^16^. Due to skewness, the Mann-Whiney test was employed to compare age, triglycerides, and the triglycerides/HDL-C ratio between men and women. Remaining comparisons were made using the student's *t*-test and the chi-square test.

The hazard ratio (HR) for CVD events among the participants above and below the TG/HDL-C sex-specific cut-off points, and with and without MetS, were also estimated in six Cox proportional hazard models using traditional CVD risk factors: 1) unadjusted; 2) adjusted for age and sex; 3) adjusted for sex, age and diabetes; 4) adjusted for sex, age and total cholesterol levels; 5) adjusted for sex, age, diabetes, total cholesterol levels, and current smoker; 6) adjusted according to sex, age, diabetes, total cholesterol levels, current smoker, diet, physical activity and body mass index (BMI).

*P* values <0.05 were considered as statistically significant.

Akaike’s information criteria (AIC), a statistical trade-off between the likelihood of a model against its complexity, were used as indicators of the goodness of fit of the model and prediction error. A lower AIC value indicates a better fit of the model^18^.

## Results

In 5430 individuals (85.9% of the baseline sample), 2784 (51.3%) women and 2646 (48.7%) men (or their relatives in case of death), could be surveyed again in order to obtain information concerning incidences of CVD events; information on the remaining inhabitants (n=893) could not be obtained because they were missed in their follow-ups.

As shown in Table 1, there was no significant difference in the baseline characteristics between the participants with and without the follow-up period, except for WC and BMI. The first CVD event, including angina pectoris, myocardial infarction, myocardial revascularization, and fatal or nonfatal stroke, was defined as the primary endpoint. There were also 36 non-cardiovascular deaths, without any differences in incidences between the high-risk subgroups and the low-risk subgroups as classified by either the TG/HDL-C ratio (HR = 1.16, 95% CI: 0.58, 2.33) or the MetS diagnosis (HR=1.09, 95% CI: 0.55, 2.15). Crude cumulative incidences of combined CVD outcomes, expressed, as the percentage of participants/10 years of follow-up, were 6.1% in the low TG/HDL-C ratio group, 12.6% in the high TG/HDL-C ratio group, 4.6% in participants without MetS and 14.7% participants with MetS. Table 2 compares the baseline CVD risk profiles of the study population, divided into low-risk and high-risk subgroups on the basis of the TG/HDL-C concentration ratio according to the Iranian cut-off point as well as the diagnostic MetS criteria. Additionally, evaluation of single CVD risk factors related to blood pressure, lipoprotein metabolism, and carbohydrate metabolism in the two high-risk subgroups were significantly greater compared to the low-risk subgroups, whether the TG/HDL-C concentration ratio or the MetS diagnostic criteria were used to identify risk. A higher percentage of women were found to be at risk for CVD based on the MetS criteria. Although a slightly greater number of high-risk individuals were identified as being at high-risk for CVD based on the elevated TG/HDL-C ratio (Iranian cut-off point) (376,14.0%) than the MetS criteria (372, 16.5%), the prevalence of subjects with CVD events that met MetS criteria was higher. In addition, the number and prevalence of diabetes were higher in participants that met the MetS criteria than when using the Iranian cut-off points. The mean of BMI, WC, systolic blood pressure (SBP), diastolic blood pressure (DBP), fasting blood sugar (FBS) and LDL were higher among the participants with MetS when comparing to the group with a high TG/HDL-C ratio.

**Table 1:** Baseline characteristics of participants “Enrolled” and “Lost” in the follow–up period

**Variable**	**Follow-up**				***P*** **value**
**Continuous variables**	**Yes, n=5430**		**No, n=893**		
	**Mean**	**SD**	**Mean**	**SD**	
Age at Baseline (yr)	51.1	11.8	50.7	11.6	0.380
BMI (kg/m^2^)	26.2	4.69	26.7	4.45	0.006
WC (cm)	92.7	12.6	94.7	12.3	0.001
SBP (mmHg)	120.7	20.9	121.6	20.9	0.239
DBP (mmHg)	78.7	11.3	78.4	11.5	0.500
Glucose (mmol/L)	86.6	27.1	88.7	32.8	0.081
Total-C (mmol/L)	212.1	52.7	214.1	52.2	0.298
LDL-C (mmol/L)	127.6	42.1	128.9	43.4	0.381
HDL-C (mmol/L)	46.4	10.5	46.9	10.4	0.165
Triglycerides (mmol/L)	190.9	108.2	191.3	103.3	0.917
**Categorical variables**	**Number**	**Percent**	**Number**	**Percent**	***P*** **values**
Women (%)	2784	51.3	471	52.7	0.414
Diabetes (%)	539	9.9	78	8.7	0.266
Current smoker	1192	22.9	206	23.1	0.458

**Table 2:** Cardiovascular disease events in follow-ups & baseline cardio-metabolic risk profile in the low-risk or high-risk subgroups based on the TG/HDL-C concentration ratio or the metabolic syndrome (MetS) diagnostic criteria

	**TG/HDL-C ratio**				***P*** **value**	**MetS**				***P*** **value**
	**Low, n=3629**		**High, n=2694**			**No, n=4066**		**Yes, n=2257**		
**Continuous variables**	**Mean**	**SD**	**Mean**	**SD**		**Mean**	**SD**	**Mean**	**SD**	
Age (yr)	50.7	12.0	50.8	11.2	0.843	49.3	11.5	53.4	11.5	0.001
BMI (kg/m2)	25.7	4.4	27.9	4.3	0.001	25.3	4.05	29.0	4.2	0.001
WC (cm)	91.9	12.3	97.8	11.4	0.001	90.3	11.4	101.9	10.1	0.001
SBP (mmHg)	119.4	20.2	124.4	21.5	0.001	115.5	17.4	132.3	22.3	0.001
DBP (mmHg)	77.3	11.2	79.9	11.8	0.001	75.4	9.9	83.7	12.3	0.001
Glucose (mmol/L)	84.5	26.4	93.6	37.8	0.001	82.0	21.6	99.9	42.9	0.001
Total-C (mmol/L)	200.8	45.9	231.4	55.1	0.001	205.2	49.1	229.4	54.3	0.001
LDL-C (mmol/L)	124.9	40.8	134.0	45.8	0.001	124.0	41.4	137.3	45.1	0.001
HDL-C (mmol/L)	50.0	10.0	42.6	9.3	0.001	48.4	10.4	44.1	9.8	0.001
Triglycerides (mmol/L)	129.8	42.0	274.0	105.2	0.001	164.1	92.9	240.0	105.0	0.001
Physical Activity	890.1	9.2	845.5	10.3	0.001	955.3	8.7	719.4	10.5	0.001
Dietary Diversity Score	4.9	1.7	5.1	1.7	0.005	4.9	1.70	5.2	1.8	0.001
**Categorical variables**	**Number**	**Percent**	**Number**	**Percent**	***P*** **value**	**Number**	**Percent**	**Number**	**Percent**	***P*** **value**
Women	1795	49.5	1460	54.2	0.001	1614	39.7	1641	72.7	0.001
CVD events	329	9.1	376	14.0	0.001	333	8.2	372	16.5	0.001
Diabetes	227	6.3	390	14.5	0.001	121	3.0	496	22.0	0.001
Current smoker	816	22.5	582	21.6	0.408	1098	27.0	300	13.3	0.001

Table 3 shows that the unadjusted HR for developing a CVD event was significantly increased in both groups designated as high-risk versus low-risk at baseline.

**Table 3:** Hazard ratios (HRs) OF CVD events in participants at baseline with a high vs. low TG/HDL-C ratio compared to participants with and without metabolic syndrome (MetS)

**Cox proportional** **hazard models**	**High vs. Low TG/HDL-C**						**MetS =Yes vs. MetS =No**		
	**Women >3.76, men >4.42** **Iranian Cut-off, n=2694**			**Women >2.5, men >3.5** **European cut-off, n=3133**			**3> criteria, n=2257**		
	**HR (95% Cl)**	***P *** **value**	**AIC**	**HR (95% Cl)**	***P *** **value**	**AIC**	**HR (95% Cl)**	***P *** **value**	**AIC**
Model 1	1.54 (1.33, 1.79)	0.001	11784	1.53 (1.31, 1.78)	0.001	11786	2.08 (1.80, 2.41)	0.001	11733
Model 2	1.58 (1.36, 1.84)	0.001	11480	1.51 (1.30, 1.76)	0.001	11487	1.99 (1.70, 2.33)	0.001	11455
Model 3	1.45 (1.24, 1.68)	0.001	11434	1.38 (1.18, 1.61)	0.001	11440	1.70 (1.43, 2.01)	0.001	11427
Model 4	1.46 (1.25, 1.71)	0.001	11471	1.39 (1.19, 1.63)	0.001	11476	1.86 (1.59, 2.19)	0.001	11443
Model 5	1.46 (1.25, 1.71)	0.001	11411	1.29 (1.11, 1.52)	0.001	11416	1.63 (1.37, 1.94)	0.001	11399
Model 6	1.32 (1.13, 1.55)	0.001	11406	1.25 (1.06, 1.47)	0.007	11411	1.51 (1.26, 1.81)	0.001	11399

Model 1: Unadjusted; Model 2: Adjusted according to sex and age; Model 3: Adjusted according to sex, age and diabetes; Model 4: Adjusted according to sex, age, and total cholesterol levels; Model 5: Adjusted according to sex, age, diabetes, total cholesterol levels and current smoker; Model 6: Adjusted according to sex, age, diabetes, total cholesterol levels, current smoker, diet, physical activity and BMI.

Although the unadjusted HR was greatest in the high-risk individuals identified by the MetS criteria (HR=2.08, 95% CI: 1.80, 2.41) compared to those identified as high-risks by the Iranian and European TG/HDL concentration ratio Cut-off points (HR=1.54, 95% CI: 1.33, 1.79 vs. HR=1.53, 95% CI: 1.31, 1.78, respectively), there are no remarkable changes after adjusting for differences in sex and age in Model 2 (HR=1.99 (1.70, 2.33), 95% CI: 1.18, 3.72 vs. HR=1.58, 95% VI: 1.36, 1.84, HR=1.51 95% CI: 1.30, 1.76, HR= for MetS, Iranian TG/HDL-C Cut-off points and European TG/HDL-C Cut-off points, respectively). Furthermore, the HR continued to be greatest in the high-risk individuals identified by the MetS criteria compared to those identified as high risks by the Iranian and European TG/HDL concentrations cut-off points after adjustments in models 3, 4, 5, and 6. Moreover, model 6 having sex, age, diabetes, total cholesterol levels, current smoker, diet, physical activity and BMI adjusted, has the smallest AIC, indicating the best fit in the model among participants with MetS.

## Discussion

Our findings indicated that MetS has a powerful predictive value for CVD events and though MetS showed to be relatively more powerful in predicting CVD events compared to the TG/HDL-C, the ratio revealed to be a powerful predictor even after adjusting for the confounding factors of age, current smoker, physical activity, family history of CVD, and BMI. TG/HDL-C could be considered to be a potential, simple tool for early identification of individuals at risk of CVD events.

Our study extends the previous findings by reporting the association between CVD events and high TG/HDL-C ratios among American, Korean, and Taiwanese populations^19-21^.

The detection of an elevated TG/HDL-C is not meant to replace the MetS diagnosis in clinical practice; rather, it should be considered as a simple tool to rapidly recognize patients that are at increased cardio metabolic risk for whom further risk evaluation and clinical intervention are needed^22^. Indeed, in young adults, compared to a MetS diagnosis, the TG/HDL-C ratio might be able to identify a greater number of individuals at risk; however, the use of a MetS diagnostic has been found to identify individuals with an accentuated cardiometabolic risk profile^20^.

While a MetS diagnosis may provide a more inclusive way to recognize the presence of cardiometabolic risk factors in individuals, this diagnostic approach is hampered by the fact that waist circumference, one of the integrative components of MetS, is not usually measured in clinical practice. Certainly, WC is normally determined by only 6% of primary care physicians^23^. This means that more than 90% of primary care physicians would not be able to diagnose individuals with MetS given that one of its components is not regularly measured. Sience TG and HDL-C are regularly measured in clinical setting, and calculating TG/HDL-C is simple for providing a fast tool to recognize cardiometabolic risk. Using TG/HDL-C to identify individuals at risk for whom further care is needed may reduce the time for and the complexity of initial diagnosis of people with cardiometabolic abnormalities^24-26^.

According to Table 3, both unadjusted and adjusted models were used to compare the ratio of TG/HDL (Iranian and European cut-off pints) with MetS. Each adjusted model includes specific well-known traditional risk factors for CVD events. In addition, the unadjusted model (model 1) compares subjects without adjusting for any of the mentioned risk factors. This strategy was used to further investigate the effective role of each risk factor and also to see the independent predictive value of both Iranian and European TG/HDL cut-off points and MetS and comparing these together.

The results in Table 3 showed that the HR of developing a CVD event was considerably greater based on the MetS criteria versus the ICS cut-off points for high levels of TG/HDL-C. Although the unadjusted HR was greater in the high-risk group with the MetS criteria as compared with the high-risk participants with an elevated TG/HDL-C ratio, that difference essentially remained when adjusted for differences in age. Furthermore, additional adjustments for sex, diabetes, total cholesterol, and current smoker had little impact, leading to the finding that the HR for the incidence of CVD events remained superior in the high risk group with the MetS criteria, yet this was reasonably comparable in the two high-risk groups. In light of these data, it seems reasonable to conclude that an elevated plasma TG/ HDL-C concentration ratio to identify individuals at high-risk for CVD, who go on to have a CVD event, could be used as an effective tool for making a diagnosis. Moreover, TG/HDL-C was shown not to be a reliable risk marker in individuals of South Asian^21^ and African American origin^26^.

Our obtained results were comparable but slightly lower than those reported ^27^which clinically assessed a large working population of Spanish men and women and reported TG/HDL-C values of 1.62 in men and 1.18 as cut-offs for identifying men and women with MetS, respectively.

Other research groups have proposed TG/HDL-C as a potential simple tool for identifying patients at increased risk for CVD. The evidence shows TG/HDL-C to be an independent predictor of future type 2 diabetes mellitus^28^ and its related micro-vascular complications^29^; coronary heart disease^29,30^; major cardiovascular events including overall death, myocarrdial infarction, and unstable angina that required revascularization^31^ and those including angina pectoris, myocardial infarction, myocardial revascularization, and fatal or nonfatal stroke^7^; and first coronary event irrespective of BMI^27^.

The TG/HDL-C ratio has been successfully used in predicting the development of diabetes, coronary heart disease, cardiovascular events, and all-cause mortality^32,30^. Furthermore, abdominal obesity, one of the MetS diagnostic criteria, varies as a function of sex and ethnicity^33^ and questions remain as to what values should be used and in what populations.

This study has several strengths. First of all, our results were obtained from a longitudinal study so the association between TG/HDL-C and CVD events could be assumed to be reliable. It investigated a population-based sample consisting of 2784 women and 2646 men, using sex-specific cut-off points as well as the incessant monitoring for CVD events and the comprehensive assessment of several lipid measures using model discrimination and fitness.

To mention the limitations of the study, there might be a misdiagnosis of cerebrovascular events, as they were identified only by phone, though a more experienced nurse and physician were involved in a second or third phone call. Moreover, numbers of cigarettes smoked per day for an individual were not considered and instead, any number of cigarettes smoked per day was counted as a current smoker in the study. In the absence of cause of death information, and given our study design, we cannot assess the path physiologic mechanism(s) that underlie this strong relationship between the TG/HDL-C ratio and the subsequent all-cause mortality, but our data suggest that subjects with high TG/HDL-C ratios should be considered at high risk of death and they should be closely followed clinically, even in the absence of obstructive coronary artery disease. Furthermore, the ICS participants were recruited from a normal population and may be representative of the general population, however, based on our results subjects having higher than the defined cut-off point for TG/HDL-C as well as those having metabolic syndrome should be considered at high risk of cardiovascular events, therefore closely followed clinically even in the absence of a diagnosed cardiovascular disease at the time of the initial visit.

## Conclusion

MetS criteria still appear to be a superior marker compared to TG/HDL-C and it works better than the lipid ratio for identifying both men and women that are at risk of CVD events. Therefore, in clinical practice, criteria related to physical examinations along with lab results of MetS become available in order to have the superior marker to identify subjects at risk of CVD events. However, in today's practice, lab tests and in particular TG and HDL are more routinely requested than physical examinations especially in developing countries and according to our study, this lipid ratio also shows to have a strong predictive value for CVD events, though MetS is still superior in predicting CVD events.

## Acknowledgements

The authors have special thanks from staffs of Isfahan Cardiovascular Research Institute for help in this study.

## Conflict of interest

None.

## Funding

The baseline survey was supported by the grant number 31309304. The undersecretary of research of the ministry of health and Isfahan cardiovascular research center, cardiovascular research institute affiliated to Isfahan University of Medical Sciences funded the ICS (NS).

## Highlights

- Triglyceride/High-Density lipoprotein cholesterol alternate cut-off points of 3.76 and 4.42 had a strong predictive value for cardiovascular disease events
- Metabolic syndrome criteria appear to be a superior marker compared to triglyceride/high-density lipoprotein cholesterol to identify patients at cardiovascular risk
- lipid ratio also shows a remarkable predictive value and could be considered to achieve this goal when appropriate
